# Microfilm Coatings: A Biomaterial-Based Strategy for Modulating Femoral Deflection

**DOI:** 10.3390/jfb15100283

**Published:** 2024-09-25

**Authors:** Ana Elisabeth Olivares-Hernandez, Miguel Angel Olivares-Robles, Juan Vicente Méndez-Méndez, Claudia Gutiérrez-Camacho

**Affiliations:** 1 Instituto Politecnico Nacional, Seccion de Estudios de Posgrado e Investigacion, Escuela Nacional de Ciencias Biologicas, Ciudad de Mexico 11340, Mexico; 2 Instituto Politecnico Nacional, Seccion de Estudios de Posgrado e Investigacion, Escuela Superior de Ingenieria Mecanica y Electrica Unidad Culhuacan, Coyoacan, Ciudad de Mexico 04430, Mexico; 3 Instituto Politecnico Nacional, Centro de Nanociencias y Micro y Nanotecnologías, “Unidad Profesional Adolfo Lopez Mateos”, Luis Enrico Erro s/n, Ciudad de Mexico 07738, Mexico; jmendezm@ipn.mx; 4 Hospital Infantil de Mexico Federico Gomez, Direccion de Enseñanza y Desarrollo Académico, Ciudad de Mexico 06720, Mexico; claudia.g.cam@facmed.unam.mx

**Keywords:** femoral head, femur coatings, microfilms, biomaterials

## Abstract

Wear on the surface of the femoral head increases the risk of hip and femur fractures. Biomechanical experiments conducted on the femur are based on its bending and torsional rigidities. Studies regarding the deflection of the femur bone when the femoral head is coated with microfilms composed of durable and compatible biomaterials are poor. This study aimed to investigate the effects of different biomaterial microfilm coatings over the femoral head on the deflection of the human femur. We utilized 2023 R1 finite element analysis (FEA) software to model the directional deformation on the femoral head and examine the femur’s deflection with varying microfilm thicknesses. The deflection of the femur bone was reported when the femoral head was uncoated and coated with titanium, stainless steel, and pure gold microfilms of different thicknesses (namely, 50, 75, and 100 μm). Our results show that the femur’s minimum and maximum deflection occurred for stainless steel and gold, respectively. The deformation of the femur was lower when the femoral head was coated with a 50-micrometer microfilm of stainless steel, compared to the deformation obtained with gold and titanium. When the thickness of the microfilm for each of the materials was increased, the deformation continued to decrease. The minimum deformation of the femur occurred for a thickness of 100 μm with stainless steel, followed by titanium and gold. The difference in the directional deformation of the femur between the materials was more significant when the coating was 100 μm, compared to the thicknesses of 50 and 75 μm. The findings of this study are expected to significantly contribute to the development of advanced medical techniques to enhance the quality of life for patients with femur bone-related issues. This information can be used to develop more resilient coatings that can withstand wear and tear.

## 1. Introduction

The femur is the main bone through which body weight is transferred to the rest of the lower limbs. Its function is to support the weight of the trunk and upper limbs and distribute it to the knee and ankle joints [1]. Several health conditions can compromise bone integrity, leading to fractures in the femur. Among the main factors are osteoporosis in 70% of non-traumatic cases [2,3,4], previous fractures in bones such as the radius of the spine [5,6], and abnormal bone architecture, or osteomalacia [7,8,9]. On the other hand, obesity has been linked to osteoporosis. However, in both pathologies, bone mineral density is affected, and it has been proven that fractures in children are associated with alterations in body composition and parameters related to bone metabolism. However, the bone–adipose tissue interaction still presents discrepancies in the literature [10,11,12].

In Mexico, the incidence of hip fractures represents a health problem in both men and women (1297 and 1725 cases per 100,000 citizens, respectively) [13]. According to global studies, the incidence of fractures secondary to osteoporosis will be at least 6.5 million yearly cases in 2050 [14]. The most common femoral fractures involve the head, femoral neck, and intertrochanteric zone, also known as the surgical neck [15].

Treatment for such fractures involves a surgical procedure to stabilize the affected segment (osteosynthesis) or joint replacement (prosthesis) [16]. These procedures can compromise health status due to secondary complications, including death within the first 24 days after surgery [13], pressure ulcers [17], thromboembolism [18], prosthetic dislocation [19], and fractures around the implant [20,21].

However, prophylactic treatments are rarely used and usually reserved for patients in whom the surgical procedure is impossible due to personal factors. Therefore, in this paper, we propose resurfacing the proximal end of the femur as a preventive treatment for fractures in this bone.

Physical methods and numerical simulations have been extensively used to study the mechanical behavior of human tissues under various conditions. Numerical simulations were used to replicate the environment and obtain realistic results due to limitations in the stress analysis of real bones.

Finite element analysis (FEA) is a promising technique based on the pre-processing of CT images to determine the geometry of human internal or external structures [22] and to study their mechanical behavior. 

In 1995, Lotz et al. reported the relationship between osteoporotic bone and the implications of presenting a fracture; they studied the stress distribution during the gait and analyzed the mechanical behavior during falls using FEA. They concluded that the stress distribution was similar for osteoporotic bone compared to normal bone; nonetheless, the peak stress increased by between 33% and 45% [23].

To assess the performance of orthopedic implants, Papini et al. [1] tested the biomechanical behavior, identifying the main stress to facilitate the design of femur implants. After studying fracture fixation procedures, Tiayne et al. [24] compared the advantages and disadvantages of internal fixation methods for neck fracture fixation. Using FEA to study mechanical behavior allowed for the analysis of different human characteristics, such as age and sex [25]. Some challenges in orthopedic treatments such as fixation procedures are the implant’s lifetime (10–15 years), risk factors in the obese population undergoing total knee arthroplasty [26], and susceptibility to thrombosis [27], among others.

Surface coating is an alternative treatment for orthopedic implants to enhance osseointegration and reduce bacterial colonization [28,29,30]. Bohara S. et al. provided an overview of potential coating technology as a proposed solution to prevent the aseptic loosening of prosthetic components. They discussed four coating materials: hydroxyapatite, collagen type I, magnesium, and chitosan. These materials provide an antibacterial function and improve osseointegration [30].

In 2020, Vogel et al. simulated a hip resurfacing arthroplasty (HRA) using different materials—such as cobalt–chromium alloy (CoCr), alumina-toughened zirconia (ATZ), and polyether ether ketone (PEEK)—to assemble a coating attached to the femoral head. They analyzed the distribution of stress and strain in the adjacent bone, concluding that stress and strain shielding in the femoral head were reduced when using a hybrid material with a PEEK body for HRA [31].

Biomaterials in orthopedic devices are used in fixation techniques to reduce the risk of rejection. Modifying the composition of polymeric molecules that produce triggered cellular responses can induce the healing process [32].

An advantage of microfilms is their implementation in various health conditions where a prosthesis is contraindicated, such as osteoporosis, due to decreased bone quality [33].

In 2023, Fada et al. studied the mechanical properties of a multicomponent scaffold with different porosity percentages. They concluded that the particle size of strontium nitrate nanoparticles with calcium phosphate improved the mechanical properties. This technique can be implemented according to the patient’s needs [34]. On the other hand, studies have been carried out on coatings based on biocompatible ultrananocrystalline diamond as a nanocoating to improve the biocompatibility of biomedical implants, where no inflammation or multinucleated giant cells were observed in the osseointegration of titanium implants [35].

This investigation aimed to avoid fractures, assess the load distribution in the proximal femur of participants without comorbidities or injuries, and determine how the thickness of the microfilm affected the modulation of femoral deflection.

To address the knowledge gap, we examined the influence of various biomaterial microfilm coatings on human femoral deflection. Utilizing finite element analysis (FEA), we modeled the directional deformation across the femoral head and analyzed deflections under varying coating thicknesses. This study simulates titanium, stainless steel, and pure gold coatings at 50, 75, and 100 μm thicknesses. 

## 2. Materials and Methods

FEA was used to study the mechanical behavior of the deformation induced in the femur under different conditions. A representative database provided geometric data for the finite element model.

### 2.1. Image Acquisition

**Figure 1:**
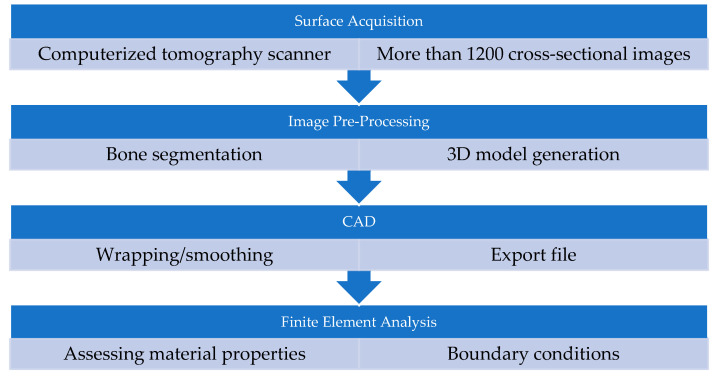
Data acquisition for human femur bone [25].

Figure 1 shows the process used to generate a 3D model of a human femur. A scanner is used to obtain computed tomography (CT) images of the femur’s surface, where an X-ray beam is aimed at the patient and quickly rotates around the body, producing signals to be processed by the machine’s computer. The patient is prone and positioned on the CT table with their feet together.

Then, all cross-sectional images are pre-processed to generate a 3D model, which is converted into .iges format to be analyzed using the 2023 R1 FEA software.

In this study, the 3D femur model was obtained from the 3D CAD model library [36], which was previously used for a research article published in 2015.

### 2.2. Model Creation and Mesh Generation

The present model was imported as a CAD solid to ANSYS Workbench, with a volume of $5.423\times 10^{5}\;{mm}^{3}\;$and a mass of 0.9761 kg. All geometric parameters, such as the ante-torsion angle, cervical–diaphyseal angle, and Sourcil index (SI) [37] (measured as the angle formed between the center of rotation of the femoral head and the medial and lateral borders of the source on plain AP radiographs), were identified in the geometry.

**Figure 2:**
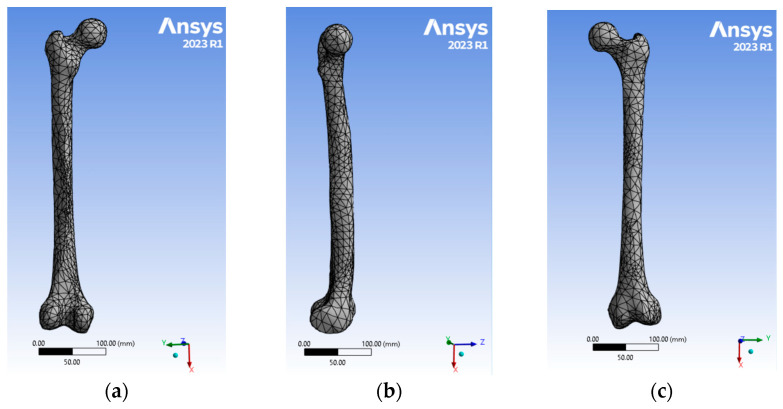
Three different views of the mesh created with the FEA software: (**a**) coronal plane showing the anterior view; (**b**) medial plane showing the lateral view; and (**c**) coronal plane showing the posterior view.

Due to the complexity of the bone architecture, this model was meshed using tetrahedral elements; the meshing used the values listed below. Figure 2 shows three views of the meshing generated in the femur model.

Number of elements = 2692

Number of nodes = 5125

We assumed orthotropic bone properties for the FEA, such as Young’s modulus, Poisson ratio, bulk modulus, shear modulus, and orthotropic stress limits assigned to the model under the static structure tool. Table 1 shows the bone’s orthotopic elasticity and orthotropic stress limits in the X, Y, and Z directions. 

### 2.3. Boundary Conditions for Femur Simulation

**Figure 3:**
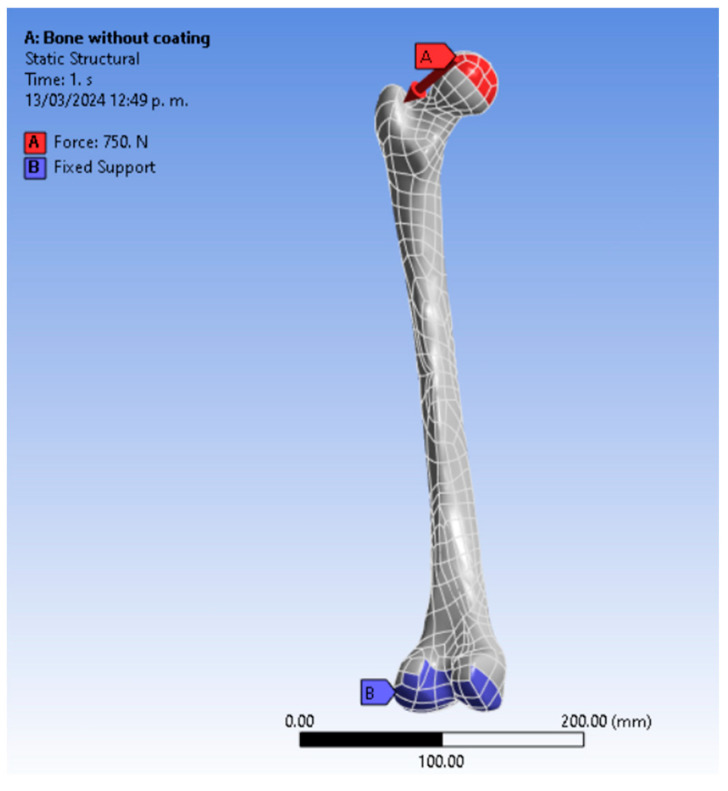
The boundary conditions are as follows: surface load in the femoral head and fixed support established in the lateral and medial femoral condyles.

The 3D femur structure was analyzed using the ANSYS 23.0 Workbench FEA software, setting the geometry as static. The directional deformation in the X, Y, and Z axes generated was investigated. For the numerical simulation, a force of 750 N was applied to the proximal epiphysis (femoral head), and the distal epiphysis of the femur bone was used as a fixed support. Figure 3 shows the femur’s boundary conditions, while the mechanical properties used are shown in Table 2.

### 2.4. Metal Coating Simulation

Several biomaterials are used in orthopedic implants, mainly metals such as titanium, cobalt–chromium alloy, gold, and stainless steel [40], considering their high corrosion resistance, suitable mechanical properties, and biocompatibility for hip prostheses [41]. Other materials used include ceramics, such as alumina, hydroxyapatite, and zirconia, and polymers, such as polymethylmethacrylate, polyether ether ketone, and polytetrafluoroethylene [40]. Ceramics and polymers have a promising future and continue to be widely studied, as they can achieve similar behavior to standard implant materials, such as stainless steel, if their mechanical properties are manipulated appropriately [42]. Titanium screws and plates did not cause more significant weight loss or metal release than single-material constructions, indicating comparable clinical safety [43].

The mechanical properties of metals, including modulus of elasticity, resistance to load, and exceptional fatigue resistance, are well documented [44]. Among other applications, their biocompatibility with human tissue has led to their widespread use in orthopedic and dental implants. The implementation of materials such as stainless steel, titanium, gold, and magnesium [45] in medical devices is primarily driven by their advantageous characteristics, including a reduced risk of implant rejection, an extended lifespan compared to other materials, and a diminished likelihood of device failure [32]. However, non-metallic materials, such as polymers, possess inherent risks, including the potential for fragmentation of the cement used to adapt to bone and osteolysis. In this study, non-biodegradable metals such as titanium, gold, and stainless steel were chosen because they have been extensively used as biomaterials for medical implant devices in recent years [46]. 

**Figure 4:**
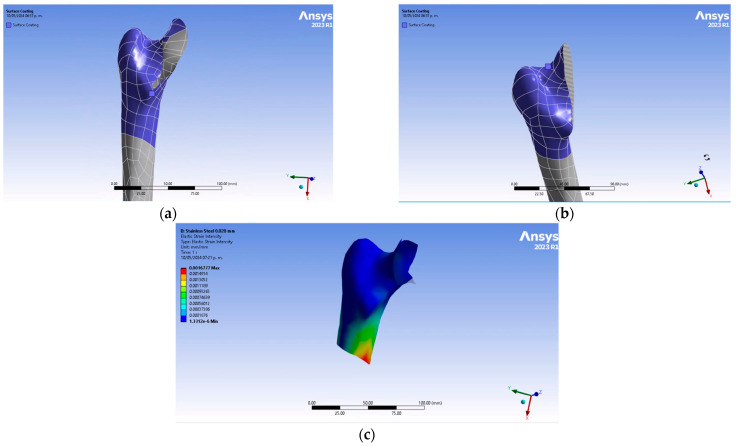
Microfilm coating attached to the femoral head: (**a**) microfilm coating shape on the bone section plane, (**b**) femur–microfilm interaction, and (**c**) stress distribution on the microfilm coating.

Biocompatible metal coatings are attached to the femoral epiphysis and proximal metaphysis. The microfilm coating is shaped to match the bone, allowing it to interact with the surface of the bone. Figure 4a–c shows the shape of the coating molded to the bone section plane and the direct contact interaction of the femur with the microfilm.

Table 3 shows the material properties. Gold, titanium, and stainless steel were the biocompatible metals used for the epiphysis coating. 

The coating thickness varied from 50 to 100 μm; all boundary conditions were preserved.

### 2.5. Deflection and Maximum Stress Formulae

The differential equation to calculate the deflection of the femur is as follows:

(1) $$\frac{\partial^{2}v}{\partial x^{2}}+\frac{P}{EI}v=-\frac{P}{EI}e$$

where *P*, *E*, *I*, *e*, and *v* are the applied load, modulus of elasticity, moment of inertia, eccentricity, and deflection, respectively.

(2) $$\sigma_{Max}=\frac{P}{A}\left(1+\frac{ec}{r^{2}}Sec\left(\frac{L}{2r}\sqrt{\frac{P}{EA}}\right)\right)$$

where $\sigma_{Max}$, r, and A are the maximum stress, radius of gyration, and cross-sectional area of the femur, respectively. In this FEA simulation, each equation was solved per node.

### 2.6. Validation of the Finite Element Simulation 

**Figure 5:**
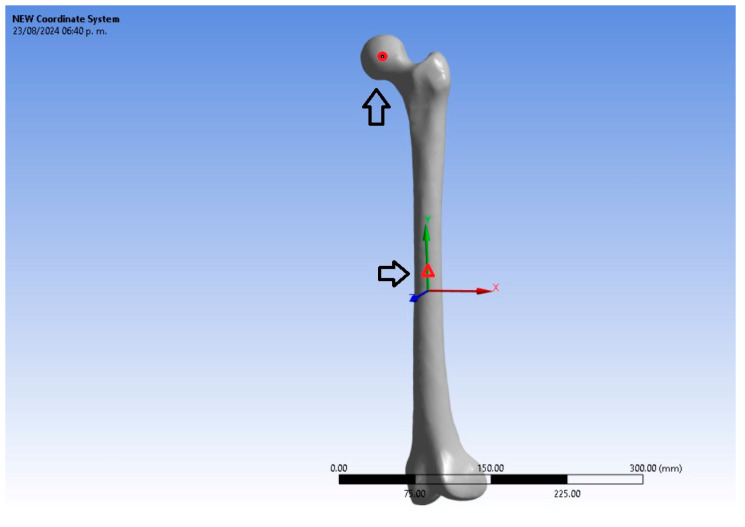
The femur shows the point’s location to evaluate vertical, Y, displacements (circle), and the point for lateral, X-Z, and displacements (triangle).

To validate our results, we followed the methodology of Pérez-Cano et al. [48]. They analyzed the load versus deformation of eight femurs. They calculated the vertical displacement of one of the points in the femoral head (circle) and the lateral displacement of a middle point of the femur, identified through the middle vertical coordinate (triangle), computed as the norm of the horizontal displacement vector, i.e., the X and Z direction. See Figure 5. Their experimental results are reported for different loads. 

### 2.7. Linear Section of the Load–Displacement Curve 

**Figure 6:**
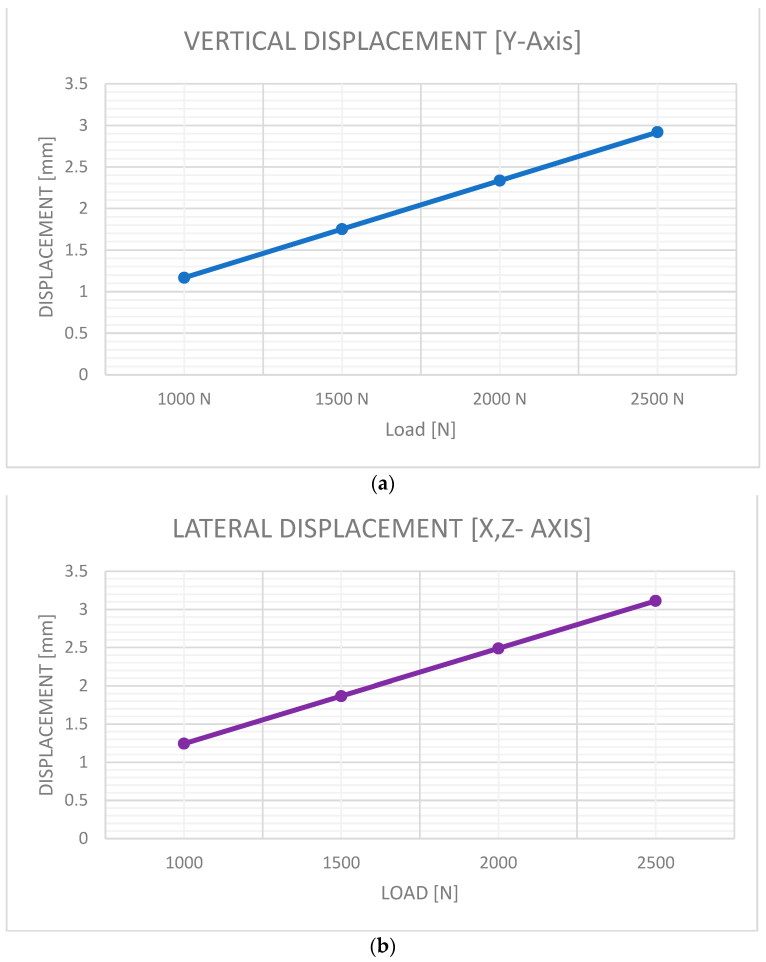
Femoral displacement under loads of 1000–2500 N, using the methodology of Pérez-Cano et al. [48]. (**a**) Vertical displacement, (**b**) Lateral displacement.

This experimental test shows that it is possible to define a linear section as 0 to 1 kN for load and 0 to 1.5 mm for vertical and lateral displacements, as shown in Figure 6a,b. This fact allows us to use a linear approximation of the experimental data in the numerical simulation of this study for vertical and lateral displacements when the load is between 0 and 1 kN. Other linear sections [49] have been defined for loads between 200 N and 400 N and displacements between 0.1 and 0.2 mm.

## 3. Results and Discussion

The results of this study were compared with those in Table 4 and Table 5, demonstrating a high level of coincidence in the findings and revealing a direct correlation between them. The results of modeling were used as control results for comparison [48].

Furthermore, Pérez-Cano et al. [48] showed that the deflection of the five femur load cells in each experimental test had a lower standard deviation for loads between 0 and 1 kN than for loads higher than 1 kN.

**Figure 7:**
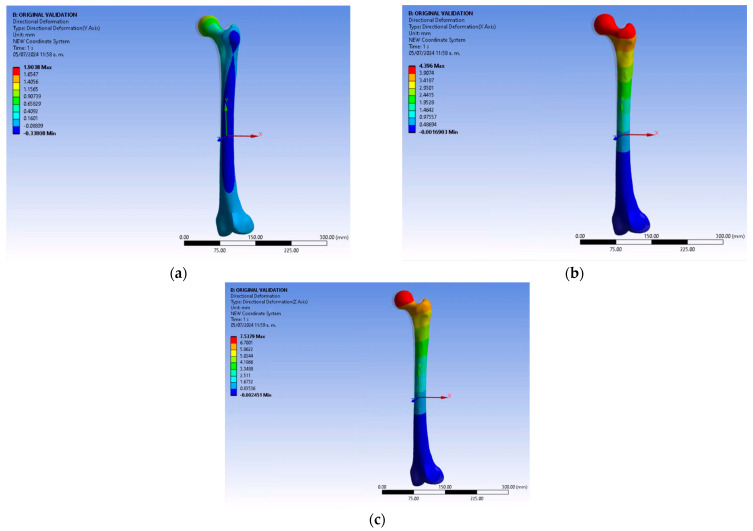
Femur behavior without coatings: (**a**) Directional deformation on the Y-axis. (**b**) Directional deformation on the X-axis. (**c**) Directional deformation on the Z-axis.

By establishing the initial conditions and entering the values of the dimensional parameters and mechanical properties of the materials, we obtained the vertical and lateral deformation of the femur. Figure 7 illustrates the deflection of the femur without any coating, serving as the baseline simulation. These data showcase both vertical and lateral deformations. Subsequent findings were compared to the behavior of an uncoated femur. The experimental conditions, including the application of load to the body, presence of fixed support, and load direction, remained unchanged without any alterations to their values. 

The uncoated femur presents the following values: vertical displacement of 0.9073 mm and lateral displacement of 0.9669 mm. These values were taken as the reference parameters. The values obtained in the X and Z axes were employed to calculate the lateral displacement. The following relation was used for each case:

$$\sqrt{x^{2}+z^{2}}$$

### 3.1. Coating Thickness of 50 µm

**Figure 8:**
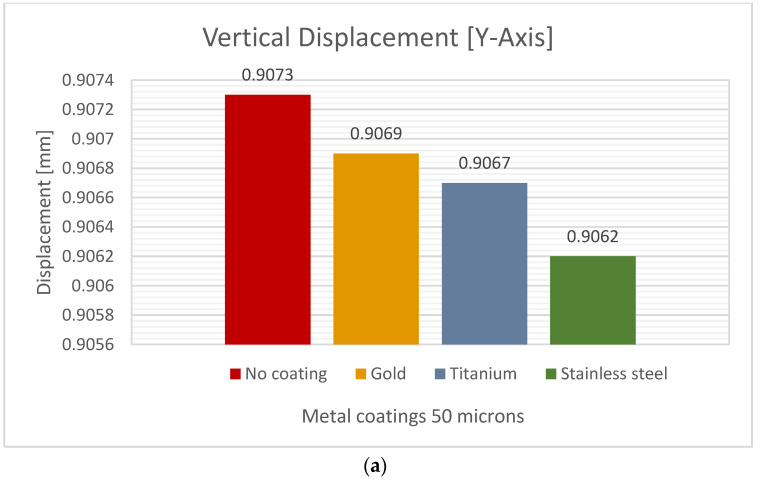

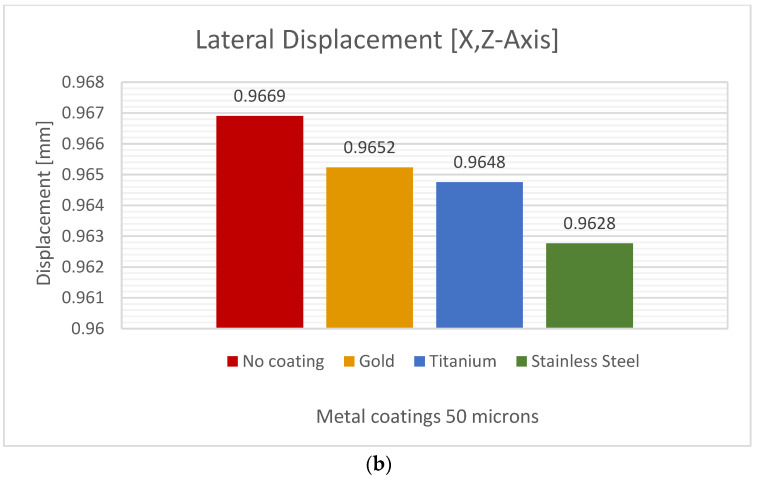
Directional deformation of the femur with a metal coating thickness of 50 µm: (**a**) vertical displacement, (**b**) lateral displacement.

Simulations were performed on 50 µm metal coatings and compared to the reference behavior. Figure 8 and Table 6 summarize the mechanical behavior of each metal coating. The first coating tested was titanium, which resulted in a maximum vertical deformation of 0.90677 mm, compared to the 0.9073 mm no-coating behavior. 

The coatings with minor deformation were stainless steel, followed by titanium, and lastly, gold. Figure 8 and Table 6 report the directional deformation. They show that the femoral lateral deformation was less with the stainless steel coating, measuring 0.9627 mm on the Y-axis. In comparison, its lateral deformation was 0.9627 mm, with stainless steel being the optimal coating material. The decrease in lateral deformation with coatings compared to uncoated deformation is shown in the last column of Table 6, Table 7 and Table 8.

Comparing titanium coating to stainless steel coating, we find a 0.066% reduction in vertical deformation. Additionally, the titanium and gold coatings exhibited similar behavior. Based on the vertical and lateral deformation behavior, a 50-micrometer-thick stainless steel coating is the best option to reduce bone deformation compared with gold and titanium coatings.

**Table 6:** Values of femur behavior (vertical and lateral deformation) with a coating thickness of 50 µm.

Material Coating Thickness 50 µm	Vertical Deformation [mm]	Lateral Deformation [mm]	Decrease in Lateral Deformation %
Stainless Steel	0.9062	0.9628	0.4344
Titanium	0.9067	0.9648	0.2275
Pure Gold	0.9069	0.9652	0.1758
No Coating	0.9073	0.9669	-

### 3.2. Coating Thickness of 75 µm

**Figure 9:**
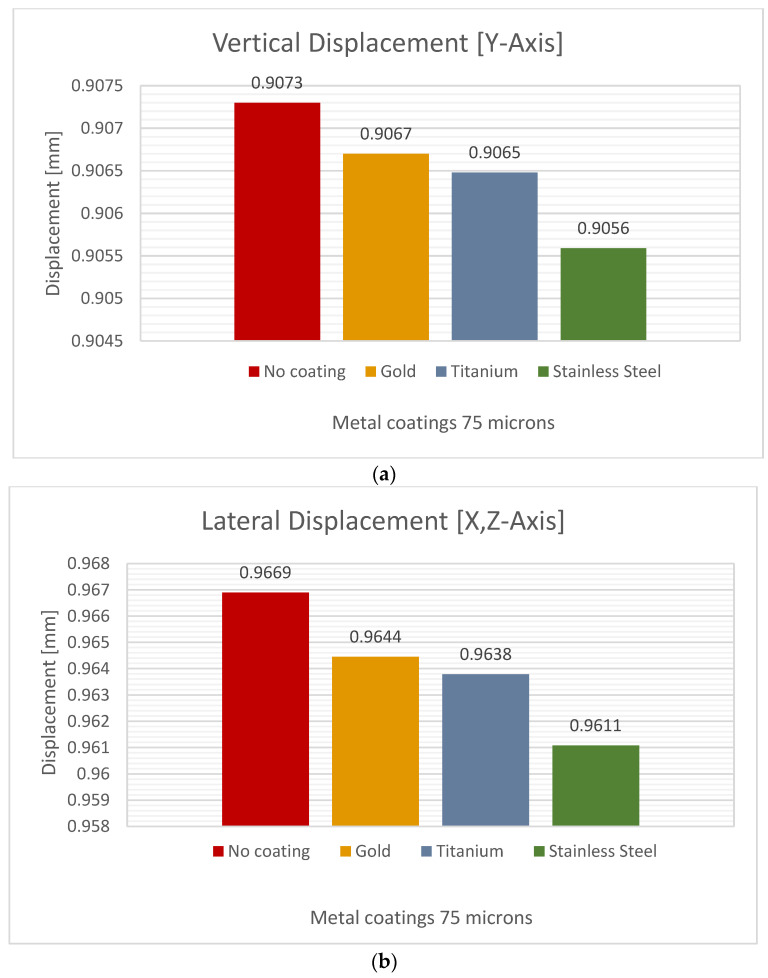
Directional deformation of the femur with a metal coating thickness of 75 µm: (**a**) vertical displacement, (**b**) lateral displacement.

In a second simulation, we varied the microfilm thickness. At a coating thickness of 75 microns, titanium was found to have the lowest lateral deformation across the femur. The gold coating also showed a minimal decrease, as shown in Figure 9 and Table 7.

**Table 7:** Values of femur behavior (vertical and lateral deformation) with a coating thickness of 75 µm.

Material Coating Thickness 75 µm	Vertical Deformation [mm]	Lateral Deformation [mm]	Decrease in Lateral Deformation %
Stainless Steel	0.9056	0.9611	0.6101
Titanium	0.9065	0.9638	0.3309
Pure Gold	0.9067	0.9644	0.2585
No Coating	0.9073	0.9669	-

We observed that the performance of stainless steel was superior to that of titanium. However, it is crucial to consider that the vertical deformation for titanium was 0.9064 mm, while for stainless steel, the maximum value was 0.9055 mm. This fact represents a 0.099% decrease in performance compared to titanium. When comparing the performance of the titanium coating to the uncoated femur, a 0.3309% decrease in lateral deformation was observed. Compared to no coating, the stainless steel coating showed a 0.6101% decrease in lateral deflection. 

### 3.3. Coating Thickness of 100 μm

**Figure 10:**
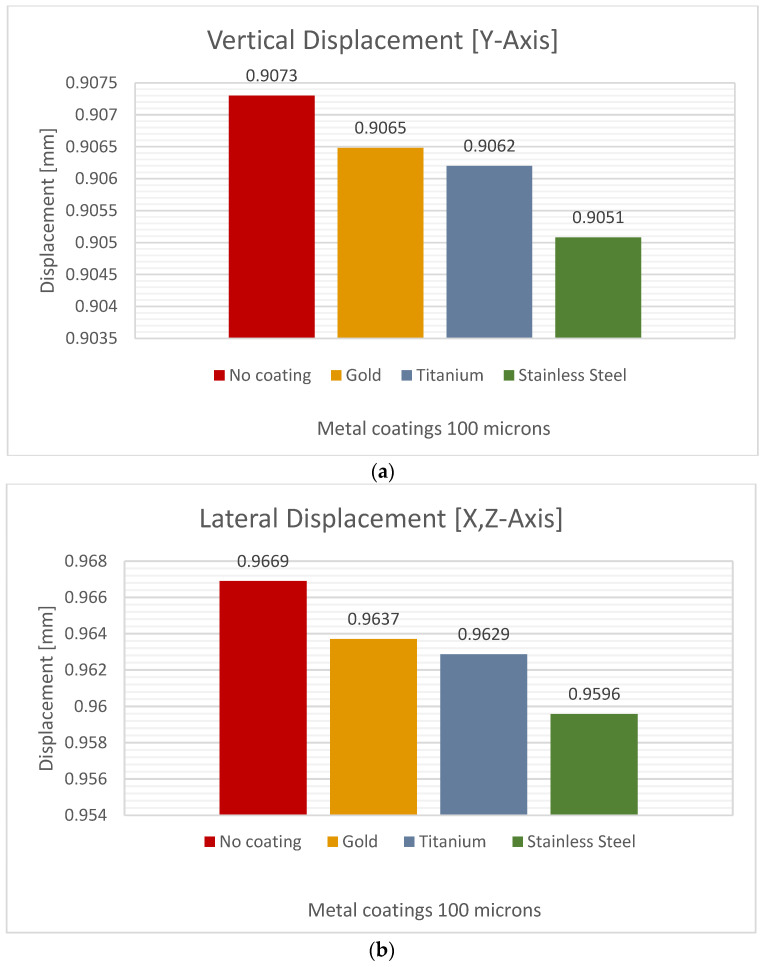
Directional deformation of the femur with a metal coating thickness of 100 µm: (**a**) vertical displacement, (**b**) lateral displacement.

Next, we considered a microfilm thickness of 100 μm. At this thickness, stainless steel showed the most minor deformation compared to titanium and gold (Figure 10). However, stainless steel exhibited 0.1324% less deformation than titanium, which had a vertical deformation value of 0.9050 mm, and the behavior of gold showed a difference of 0.99% when compared to the uncoated femur, as shown in Table 8. 

**Table 8:** Values of femur behavior (vertical and lateral deformation) with a coating thickness of 100 µm.

Material Coating Thickness 100 µm	Vertical Deformation [mm]	Lateral Deformation [mm]	Decrease in Lateral Deformation %
Stainless Steel	0.9051	0.9596	0.7653
Titanium	0.9062	0.9629	0.4240
Pure Gold	0.9065	0.9637	0.3309
No Coating	0.9073	0.9669	-

## 4. Discussion

**Figure 11:**
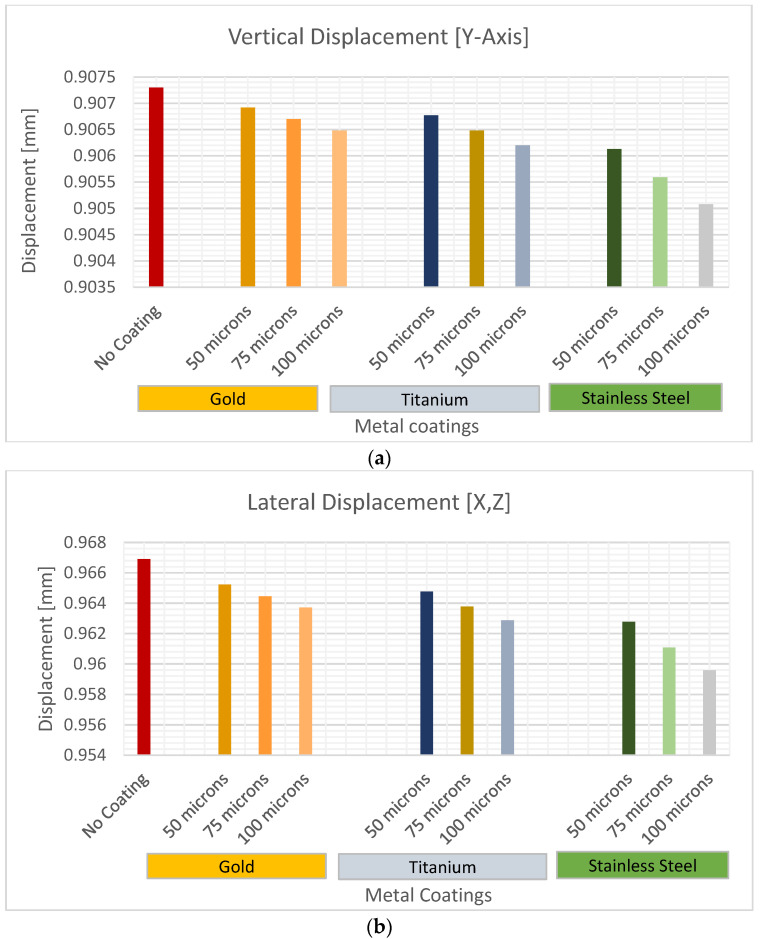
Femur deformation varying coating thickness. (**a**) vertical displacement, (**b**) lateral displacement.

This study proposes the implementation of a coating not only in the femoral head but also in the subtrochanteric region. Figure 11 compares the displacements of the femur between different materials at different thicknesses. In all cases, coating the bone with microfilms decreases bone deflection. As the thickness of the microfilm increases, there is a lower deflection of the femur for all materials used. Therefore, it is crucial to consider the appropriate thickness of the right material. Among the materials, gold limits femur deflection to a lesser extent compared to titanium and stainless steel, with stainless steel as the material that presents the greatest resistance to deflection.

The data demonstrate an interesting pattern of similarity in femur deflection when using different materials with varying thicknesses. For instance, the femur deflection achieved with a 100-micron-thick titanium microfilm closely resembles that obtained with a 50-micron-thick stainless steel coating. Similarly, the comparison between a 100-micron-thick gold microfilm and a 75-micron-thick titanium coating also reveals a comparable femur deflection. These findings suggest that it is possible to achieve consistent femur deflection by adjusting the thickness of the coating, irrespective of the material used. The difference in the vertical and lateral deformation of the femur between the materials was more significant when the coating was 100 μm thick, compared to thicknesses of 50 and 75 μm. The vertical and lateral displacement of the femur showed no significant difference between coatings of 50 and 75 microns in gold, while it was slightly different in titanium. 

The current study, involving finite element simulation, aims to provide valuable insights into improving bending reduction and stress distribution in the femoral neck using thin films. As far as we know, similar studies are lacking. Nevertheless, multiple analyses investigate stress fields under various conditions.

Numerous studies [50,51,52] investigated simulated finite element (FE) models featuring resurfaced femoral heads. These studies have demonstrated a direct relationship between the reduction in deformation in the femoral head and decreased stresses. Notably, the analysis of deflection in the femur bone has not been explored by any of the aforementioned authors, unlike the focal point of our current study.

A study conducted by Vogel et al. [31] rigorously examined a hybrid hip replacement implant consisting of Polyetheretherketone (PEEK) and a ceramic-bearing surface. Their research clearly established that the thickness of the ceramic component plays a pivotal role in determining the distribution of stress and strain. It was conclusively demonstrated that maintaining a thickness of 0.5 mm resulted in a stress and strain distribution similar to that of the PEEK implant. Any increase in coating thickness was shown to diminish the benefits. The study definitively identified the optimal coating thickness and the most suitable material for achieving the desired stress and strain distribution.

Notably, the present study specifically focused on analyzing femur deflection in response to the application of microfilms of varying biomaterial thicknesses to the femoral head and subtrochanteric region. The robust findings unequivocally show that both the material type and microfilm thickness exert a tangible influence on femur deflection. Furthermore, it was incontrovertibly demonstrated that similar femur deflection can be attained by precisely adjusting the thickness of different materials. These results robustly align with the findings of Vogel’s study.

One effective approach for implementing a metal bone coating involves using hip resurfacing surgery. This method includes the preservation and reshaping of the femoral head, followed by the application of a smooth metal cap. Additionally, the damaged bone and cartilage within the hip socket are carefully removed and replaced with a metal shell, mirroring the conventional approach utilized in a total hip replacement procedure. Thus, it is anticipated that the insights derived from this analysis will lead to advancements in the development and interpretation of non-invasive methodologies for accurately quantifying the risk of hip fractures in vivo.

It points out some practical potential and benefits of the obtained modeling results. Metallic microfilm coatings offer a superior advantage over ceramic materials by effectively reducing the risk of infection through the exceptional antibiotic properties bonded to the femoral head. It has now been reported that obese patients undergoing total knee arthroplasty significantly increase hospital costs due to several factors, including wound-healing complications, thromboembolic events, and deep infection due to the substantial amount of adipose tissue. According to Martin et al., body mass index (BMI) is significantly associated with increased re-operation rates as a complication of treatment [53]. Osteoporosis is another health condition that may benefit from the use of microfilms. Our results showed a micrometric decrease in femoral deformation. In patients with low bone mineral density, such as osteoporosis patients, this could prevent the occurrence of microfractures [54].

The long-term stability of metal coatings allows them to integrate with bone effectively, and their biocompatibility and safety produce low interaction risks between the metal coating and human bone [41]. Long-term patient outcomes include prevention of microfractures, reduction in bone surface osteoarthritis, and improved bone quality.

## 5. Conclusions

The novelty of this study is a comparison of different metal coatings on the femoral head by measuring the mechanical behavior with parameters that are difficult to measure experimentally, such as directional deformation within the bone.

Our results demonstrated that stainless steel coatings yielded the lowest femoral deflection, while gold exhibited the highest when the thickness was 100 µm. Deflection consistently decreased for all materials with an increasing coating thickness. The 100 μm coating revealed the most pronounced differences in directional deformation between the materials.

The minimum deformation of the femur for a thickness of 100 μm occurred with stainless steel, followed by titanium, and then gold. The difference in directional deformation of the femur between the materials was more significant when the coating was 100 μm, compared to the thicknesses of 50 and 75 μm. These findings offer valuable insights into the potential of microfilm coatings for mitigating femoral deflection. This research could inform the development of more resilient coatings, ultimately enhancing the durability of joint replacements and improving the quality of life for patients with femur-related conditions.

This study did not analyze the effects of surrounding muscles and ligaments on the femur.

For future research, we strongly suggest the following:

- New biomaterials: biocomposites such as hydroxyapatite or metal alloys could be used. Researchers can explore and discover new human tissue-compatible materials to reduce femoral head loading and prevent fractures.
- Health conditions: researchers should simulate different health conditions that may be potential risk factors and compromise bone continuity, such as obesity, osteopenia, or osteoporosis.
- Activities of daily living: researchers should analyze the femur under ambient conditions such as walking, running, and jumping.

## Figures and Tables

**Table 1:** Bone orthotropic properties [38].

Orthotropic Elasticity		Value	Unit
Young’s Modulus	X direction	19,400	MPa
	Y direction	12,600	MPa
	Z direction	12,600	MPa
Poisson Ratio	XY	0.39	
	YZ	0.3	
	XZ	0.9	
Shear Modulus	XY	5700	MPa
	YZ	4850	MPa
	XZ	5700	MPa
**Orthotropic Stress Limits**			
Tensile	X direction	135	MPa
	Y direction	50	MPa
	Z direction	50	MPa
Compressive	X direction	−250	MPa
	Y direction	−50	MPa
	Z direction	−50	MPa
Shear	XY	65	MPa
	YZ	65	MPa
	XZ	65	MPa

**Table 2:** Femur density and dimensions.

Mechanical Properties	Symbol	Value
Density	D	$1800\;kg/m^{3}$
Length	L	46.77 cm
Load ^1^	F	777 N

^1^ The load value was considered a walking load [39].

**Table 3:** Metals’ mechanical properties [47].

**Gold**	**Symbol**
Elasticity Modulus	7.576 × 10^10^ Pa
Poisson Ratio	0.42
Density	19,300 kg m^−3^
Thickness	50, 75, 100 µm
**Titanium**	**Symbol**
Elasticity Modulus	9.6 × 10^10^ Pa
Poisson Ratio	0.36
Density	4620 kg m^−3^
Thickness	50, 75, 100 µm
**Stainless Steel**	**Symbol**
Elasticity Modulus	2 × 10^11^ Pa
Poisson Ratio	0.3
Density	7850 kg m^−3^
Thickness	50, 75, 100 µm

**Table 4:** Validation of vertical displacements.

Load [N]	Simulation [mm]	Experimental Range [mm] [48]
777	0.9073	0.9
1000	1.1678	0.9–1.5
1500	1.7517	1.3–1.9
2000	2.3356	1.5–2.3
2500	2.9195	1.6–3.2

**Table 5:** Validation of lateral displacements.

Load [N]	Simulation Value [mm]	Experimental Range [mm] [48]
777	0.9669	1.0
1000	1.2443	0.45–1.6
1500	1.8666	1.1–2.0
2000	2.4888	1.6–2.4
2500	3.1110	1.7–2.9

## Data Availability

The original contributions presented in the study are included in the article, further inquiries can be directed to the corresponding authors.
